# Monitoring cytosolic and ER Zn^2+^ in stimulated breast cancer cells using genetically encoded FRET sensors†
†Electronic supplementary information (ESI) available. See DOI: 10.1039/c5mt00257e



**DOI:** 10.1039/c5mt00257e

**Published:** 2016-01-07

**Authors:** Anne M. Hessels, Kathryn M. Taylor, Maarten Merkx

**Affiliations:** a Laboratory of Chemical Biology and Institute of Complex Molecular Systems (ICMS) , Department of Biomedical Engineering , Eindhoven University of Technology , Eindhoven , The Netherlands . Email: M.Merkx@tue.nl; b Breast Cancer Molecular Pharmacology Group , School of Pharmacy and Pharmaceutical Sciences , Cardiff University , Cardiff , UK

## Abstract

The Zn^2+^-specific ion channel ZIP7 has been implicated to play an important role in releasing Zn^2+^ from the ER. External stimulation of breast cancer cells has been proposed to induce phosphorylation of ZIP7 by CK2α, resulting in ZIP7-mediated Zn^2+^ release from the ER into the cytosol. Here, we examined whether changes in cytosolic and ER Zn^2+^ concentrations can be detected upon such external stimuli. Two previously developed FRET sensors for Zn^2+^, eZinCh-2 (*K*_d_ = 1 nM at pH 7.1) and eCALWY-4 (*K*_d_ = 0.63 nM at pH 7.1), were expressed in both the cytosol and the ER of wild-type MCF-7 and TamR cells. Treatment of MCF-7 and TamR cells with external Zn^2+^ and pyrithione, one of the previously used triggers, resulted in an immediate increase in free Zn^2+^ in both cytosol and ER, suggesting that Zn^2+^ was directly transferred across the cellular membranes by pyrithione. Cells treated with a second trigger, EGF/ionomycin, showed no changes in intracellular Zn^2+^ levels, neither in multicolor imaging experiments that allowed simultaneous imaging of cytosolic and ER Zn^2+^, nor in experiments in which cytosolic and ER Zn^2+^ were monitored separately. In contrast to previous work using small-molecule fluorescent dyes, these results indicate that EGF–ionomycin treatment does not result in significant changes in cytosolic Zn^2+^ levels as a result from Zn^2+^ release from the ER. These results underline the importance of using genetically encoded fluorescent sensors to complement and verify intracellular imaging experiments with synthetic fluorescent Zn^2+^ dyes.

## Abstract 2

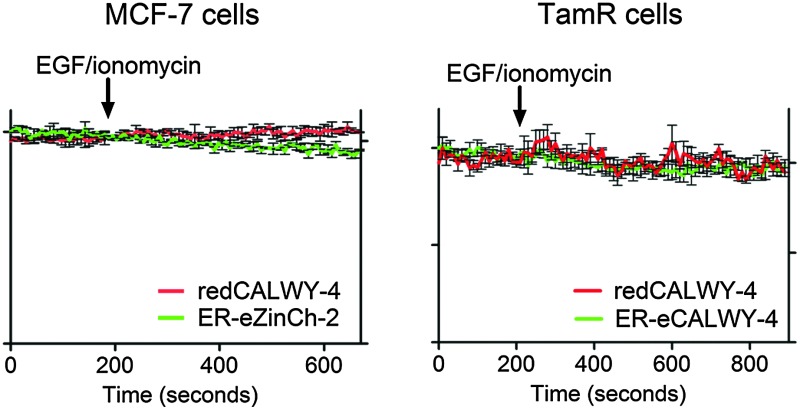
Unexpectedly, monitoring cytosolic and ER Zn^2+^ using FRET sensor proteins does not support EGF–ionomycin-triggered Zn^2+^ waves in breast cancer cells.

## Introduction

Zn^2+^ is essential for proper cell function and cell growth.^1^ However, excess Zn^2+^ can be toxic to cells, affecting growth and causing serious metabolic disorders.^2^ How Zn^2+^ participates in controlling cellular processes is an emerging area of interest. In addition to its well established roles as a Lewis acid cofactor in enzyme catalysis and its structural role in protein folding, several studies have recently proposed a role for Zn^2+^ as a secondary messenger in intracellular signal transduction pathways.^3–5^ Fluorescent sensors that allow intracellular imaging of Zn^2+^ concentrations in living cells have proven crucial in our understanding of the molecular mechanisms of intracellular Zn^2+^ homeostasis and its putative signaling role.

Whereas small molecule sensors continue to contribute valuable insights into the role of Zn^2+^ in numerous biological processes, their intracellular concentration and localization cannot be easily controlled. Therefore, we and others have developed fluorescent Zn^2+^ sensor proteins based on the modulation of Förster Resonance Energy Transfer (FRET) between a donor and acceptor fluorescent domain.^6–8^ These genetically-encoded sensors do not require cell-invasive procedures, their concentration and intracellular location can be tightly controlled, and the use of FRET allows for ratiometric detection. The eCALWY-sensors that were developed by our group consist of two small metal binding domains, ATOX1 and WD4, connected by a long flexible linker, with self-associating variants of cerulean and citrine fluorescent domains fused to the N- and C-terminus, respectively. A toolbox of 6 eCALWY variants has been developed that range in Zn^2+^ affinity between 2 pM and 5 nM. Of these, eCALWY-4 with a *K*_d_ of 630 pM was found to be optimal for measuring cytosolic Zn^2+^ concentrations, which typically reside in the 0.2–1 nM range, in a wide variety of cells.^6,9^ Replacement of cerulean and citrine by self-associating variants of mOrange and mCherry yielded the red-shifted redCALWY variants, whose spectral properties allowed them to be used in combination with CFP-YFP-based reporters.^10^ Recently, we also reported another versatile FRET sensor containing a *de novo* Cys_2_His_2_ Zn^2+^ binding pocket created directly on the surface of cerulean and citrine domains.^11^ This eZinCh-2 sensor displays a large increase in emission ratio upon binding Zn^2+^ at the interface of the two fluorescent domains, with an affinity that is similar to that of eCALWY-4. By default eCALWY-4 and eZinCh-2 reside in the cytosol, but both have been successfully targeted to the ER, mitochondria and secretory vesicles by the introduction of organelle specific targeting sequences.^11^

Because Zn^2+^ is unable to passively diffuse across cell membranes, active transport between the cytosol, the extracellular space and intracellular compartments is required to allow control over intracellular Zn^2+^ homeostasis. Two families of mammalian Zn^2+^ transporters are known that transport Zn^2+^ across cellular membranes. The ZnT (SLC30A) family exports Zn^2+^ from the cytosol into organelles or to the extracellular space.^12^ The ZIP family (SLC39A) acts as an importer protein and is responsible for Zn^2+^ import from either the extracellular space or from different cellular compartments.^13^ One of the ZIP family members, ZIP7, was found to localize on the membrane of the endoplasmic reticulum^14,15^ and is believed to play a role in the transportation of Zn^2+^ from the ER into the cytosol. Zn^2+^ transporters play a crucial role in maintaining the delicate balance between cell growth and cell death and aberrant functioning of Zn^2+^ transporters has been linked to several disease states including cancer. For example, Zn^2+^ was found to be increased in human breast tumors.^16,17^ A widely used model to investigate the development of breast cancer^18^ showed an up to 19-fold increase in total Zn^2+^ levels in mammary tumors in mice, rats and humans.^19^ One mechanism by which increased levels of cytosolic Zn^2+^ could enhance cell proliferation is by the inhibition of protein tyrosine phosphatases, which could lead to a net increase in phosphorylation of well-known cancer associated downstream effectors such as AKT and MAPK (mitogen-activated protein kinase).^20,21^

Taylor and coworkers have proposed that phosphorylation of ZIP7 might induce release of Zn^2+^ from the ER to the cytosol in Tamoxifen-resistant MCF-7 breast cancer cells (TamR).^22^ Using the membrane-permeable Zn^2+^-specific indicator Newport Green DCF a 2-fold higher fluorescence was observed in TamR cells compared to wild-type MCF-7 cells, suggesting a higher intracellular free Zn^2+^ concentration.^22^ Comparison of the expression levels of several Zn^2+^ importers (ZIPs) revealed a significant increase in expression level of ZIP7 in TamR cells compared to wild-type MCF-7 cells. Since ZIP7 is almost exclusively localized on the ER membrane,^14^ ZIP7 has been implicated to control the release of Zn^2+^ from the ER to the cytosol. Two types of triggers were used to study the involvement of ZIP7 in Zn^2+^ signaling in both wild-type MCF-7 and TamR cells: the addition of extracellular Zn^2+^ with pyrithione, and addition of EGF with ionomycin. Addition of external Zn^2+^ and pyrithione to MCF-7 and TamR cells loaded with the Zn^2+^-specific fluorescent indicator FluoZin-3 showed an increase in green fluorescence within a few minutes after stimulation, which was interpreted to be a result of ZIP7-dependent Zn^2+^ release from the ER.^21,22^ Immunoprecipitation experiments with a ZIP7 antibody in Zn^2+^ treated cells showed co-immunoprecipitation of CK2α, suggesting that this kinase may be responsible for phosphorylation of ZIP7. Consistent with this role, a peak in the physical association between protein kinase CK2α and ZIP7 was observed within two minutes after treatment with Zn^2+^/pyrithione. In addition, a proximity ligation assay, in which fluorescent dots appear when two molecules are in close proximity, showed significant association of ZIP7 and CK2α in the same timeframe.

Based on the above results a model was proposed whereby the phosphorylation of ZIP7 by CK2 results in ZIP7-mediated Zn^2+^ release from ER stores into the cytosol, resulting in subsequent activation of several downstream signaling pathways.^22^ However, the evidence for Zn^2+^ release from the ER upon external stimuli has remained indirect, because these studies did not directly measure ER Zn^2+^ levels. In addition, FluoZin-3 and the other synthetic fluorescent sensors that were employed to monitor intracellular Zn^2+^, are known to not only localize in the cytosol, but also to different cellular compartments, such as the Golgi, synaptic vesicles and the nucleus.^23^ Here, we report the use of two previously developed FRET sensors for Zn^2+^ (eZinCh-2 (*K*_d_ = 1 nM) and eCALWY-4 (*K*_d_ = 0.63 nM)) to test the hypothesis that Zn^2+^ is released from the ER into the cytosol upon external stimuli (Table 1). Unlike synthetic fluorescent sensors, these genetically encoded sensors can be targeted exclusively to either the cytosol or the ER. Moreover, the use of two different color variants allowed us to simultaneously monitor the free Zn^2+^ concentration in the cytosol and ER within the same cell. Using these sensors an immediate increase in both cytosolic and ER Zn^2+^ levels was observed upon treatment of MCF7 and TAMR cells with Zn^2+^/pyrithione, which is consistent with direct, pyrithione-mediated exchange of Zn^2+^ between the cell exterior and intracellular compartments. In contrast, addition of EGF/ionomycin did not induce significant changes in cytosolic or ER Zn^2+^ concentrations. The implication of these findings for the putative role of ZIP7 in controlled release of Zn^2+^ from the ER will be discussed.

**Table 1:** Overview of FRET sensors used in this study

Sensor	Fluorescent domains (donor/acceptor)	*K*_d_ for Zn^2+^ (pH 7.1) (nM)	Cellular localization
eZinCh-2^11^	Cerulean/citrine^*a*^	1.0	Cytosol
ER-eZinCh-2^11^	Cerulean/citrine	1.0	ER^*c*^
eCALWY-4^6^	Cerulean/citrine	0.63	Cytosol
ER-eCALWY4^24^	Cerulean/citrine	0.63	ER^*c*^
redCALWY-4^10^	mOrange2/mCherry^*b*^	0.24	Cyotosol

^*a*^Cerulean: excitation maximum at 435 nm, emission maximum at 475 nm; citrine: excitation maximum at 510 nm, emission maximum at 535 nm.

^*b*^mOrange2: excitation maximum at 549 nm, emission maximum at 565 nm; mCherry: excitation maximum at 587 nm, emission maximum at 610 nm.

^*c*^ER-targeted sensor variants contained an N-terminal insulin signal peptide and a C-terminal KDEL retention sequence.

## Results

### Determination of cytosolic and ER Zn^2+^ concentrations in MCF-7 and TamR cells using FRET-based Zn^2+^ sensors

**Fig. 1:**
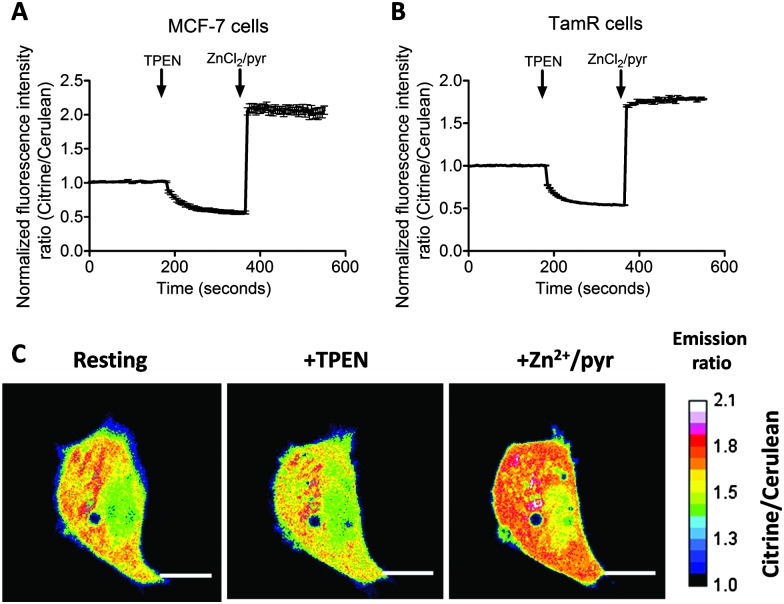
(A and B) Determination of the free cytosolic Zn^2+^ concentration in wild-type MCF-7 (A) and TamR cells (B) using eZinCh-2. Responses to the addition of 50 μM TPEN, followed by the addition of excess 100 μM Zn^2+^/ 5 μM pyrithione. All traces in A and B represent the average of at least four cells after normalization of the emission ratio at *t* = 0 s. Error bars represent the standard error of the mean (SEM). (C) False-colored ratiometric images of an MCF-7 cell expressing eZinCh-2 in resting state (resting), after perfusion with 50 μM TPEN (+TPEN), and 100 μM ZnCl_2_/5 μM pyrithione (+Zn^2+^/pyr). Scale bar, 10 μM.

Previous work on MCF-7 and TamR cells using small-molecule fluorescent sensors reported increased levels of intracellular Zn^2+^ in TamR cells compared to wild-type MCF-7 cells,^22^ but the concentration of free cytosolic Zn^2+^ could not be determined. We therefore expressed the eZinCh-2 sensor in the cytosol of both MCF-7 and TamR cells and monitored the *in situ* response in single living cells to the addition of the strong membrane-permeable Zn^2+^ chelator TPEN and excess Zn^2+^ together with pyrithione as a Zn^2+^-specific ionophore. In both MCF-7 and TamR cells a robust, 3-fold change in emission ratio was observed between the Zn^2+^-depleted and Zn^2+^-saturated states of the sensor (Fig. 1). The determination of the emission ratios under conditions of Zn^2+^ depletion (*R*_0%_) and Zn^2+^ excess (*R*_100%_) allowed calculation of the sensor occupancy under resting conditions, which could be translated into a free Zn^2+^ concentration using the *K*_d_ of 1 nM. The free cytosolic Zn^2+^ concentrations in wild type MCF-7 cells and TamR cells were determined to be 0.44 ± 0.06 nM and 0.65 ± 0.06 nM, respectively. Although the free Zn^2+^ concentration in TamR cells appears to be slightly higher, both numbers agree well with values determined previously in other mammalian cell types, and neither of them is particularly high.^6^

**Fig. 2:**
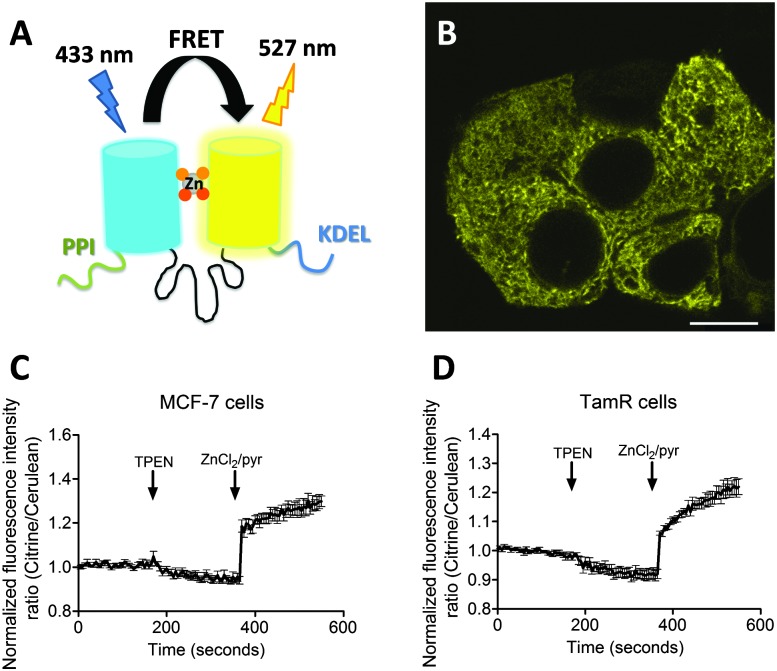
Zn^2+^ imaging using ER-targeted eZinCh-2 in both MCF-7 and TamR cells. (A) Schematic representation of eZinCh-2 containing an N-terminal signal sequence (PPI) and a C-terminal retention sequence (KDEL). (B) False-colored image showing MCF-7 cells expressing ER-eZinCh-2. (C and D) Responses of MCF-7 (C) and TamR (D) cells expressing ER-eZinCh-2 to the addition of 50 μM TPEN, and the subsequent addition of excess Zn^2+^ together with pyrithione (100 μM/5 μM). All traces represent the average of four cells after normalization of the emission ratio at *t* = 0 s. Error bars represent SEM.

**Fig. 3:**
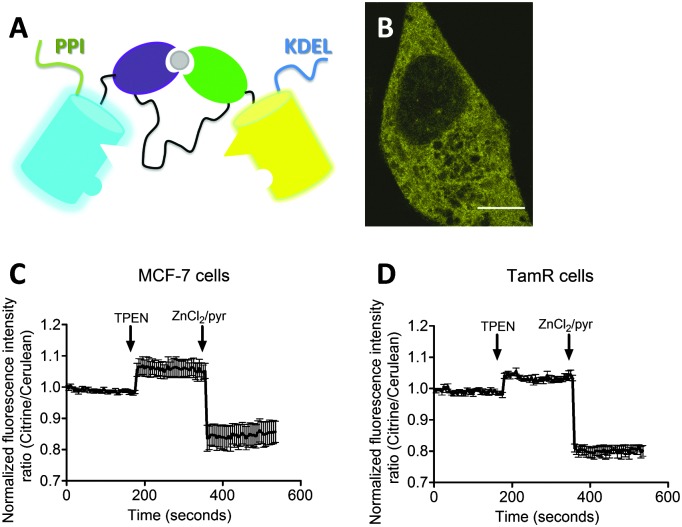
Zn^2+^ imaging using ER-targeted eCALW-4 in both MCF-7 and TamR cells. (A) Schematic representation of eCALWY-4 containing a PPI and a KDEL sequence. (B) False-colored image showing a TamR cells expressing ER-eCALWY-4. (C and D) Responses of MCF-7 (C) and TamR (D) cells expressing ER-eCALWY-4 to the addition of 50 μM TPEN, and the subsequent addition of Zn^2+^/pyrithione. All traces in represent the average of four cells after normalization of the emission ratio at *t* = 0 s. Error bars represent SEM.

To explore a possible correlation between the increased ZIP7 expression and free ER Zn^2+^ levels in TamR cells,^22^ the ER-targeted FRET-based Zn^2+^ sensors ER-eZinCh-2 and ER-eCALWY-4 were expressed in both wild-type MCF-7 and TamR cells^24^ (Table 1 and Fig. 2 and 3). Colocalization studies showed exclusive localization of these sensors to the ER lumen. Using ER-eZinCh-2, addition of TPEN and excess Zn^2+^ yielded similar response curves as were found previously for HeLa and HEK293T cells,^11^ yielding free ER Zn^2+^ concentrations of 0.54 ± 0.27 nM for MCF-7 (Fig. 2C) and 0.75 ± 0.49 nM for TamR cells (Fig. 2D). Measurements using ER-targeted eCALWY-4 yielded similar ER Zn^2+^ concentrations of 0.39 ± 0.17 nM and 0.21 ± 0.05 nM for MCF-7 (Fig. 3C) and TamR cells (Fig. 3D), respectively.

The average free ER Zn^2+^ concentrations that were found are similar to those found in the cytosol, although the cell-to-cell variation in free Zn^2+^ concentration was found to be larger in the ER when compared to the cytosol. The latter may reflect less efficient buffering of the free Zn^2+^ concentration due to a lack of metallothionein in the ER.

### Monitoring changes in intracellular Zn^2+^ levels upon external Zn^2+^ stimulation

**Fig. 4:**
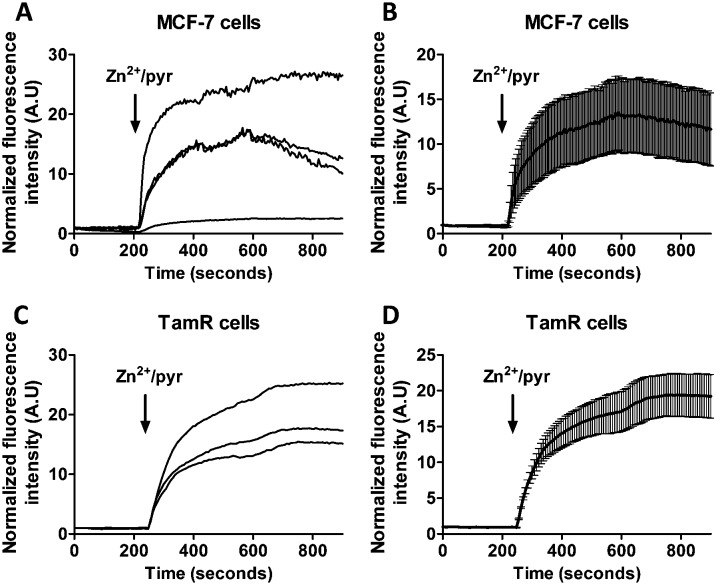
(A and B) Responses of MCF-7 cells loaded with FluoZin-3 AM to the addition of 20 μM Zn^2+^ together with the Zn^2+^-specific ionophore pyrithione, monitored in real time. (C and D) Responses of TamR cells loaded with FluoZin-3 AM to the addition of 20 μM Zn^2+^ together with the Zn^2+^-specific ionophore pyrithione, monitored in real time. Traces in B and D represent the average of four and three cells, respectively, after normalization at *t* = 0 s. Error bars represent SEM.

Previous studies using FluoZin-3 showed a delayed increase in intracellular Zn^2+^ upon extracellular addition of 20 μM Zn^2+^ and 10 μM pyrithione, which was interpreted to result from ZIP7-dependent Zn^2+^ release from ER stores into the cytosol.^21^ Because pyrithione is normally used to transfer Zn^2+^ ions across biological membranes, one would expect to observe a large and immediate increase in intracellular Zn^2+^ levels upon Zn^2+^/pyrithone treatment, but the cells in the previous study were fixed prior to imaging. To check whether the same results could be obtained in live cells, we repeated these imaging experiments on live MCF-7 and TamR cells loaded with FluoZin-3 AM. A perfusion setup was used to allow continuous flow over the cells during the imaging experiments. In this experimental setup, addition of external Zn^2+^ resulted in a large increase in fluorescence immediately after addition of 20 μM Zn^2+^/10 μM pyrithione for both wild-type MCF-7 (Fig. 4A and B) and TamR cells (Fig. 4C and D). A large variation in absolute fluorescent signal intensity was observed after Zn^2+^ addition between individual cells, which may reflect the difficulty to control the intracellular concentration of FluoZin-3. Nonetheless, in contrast to previous work using fixed cells, these live cell imaging experiments reveal an almost immediate increase in fluorescence intensity in all cells with a half-time of about 60 seconds after exposure to Zn^2+^/pyrithione.

**Fig. 5:**
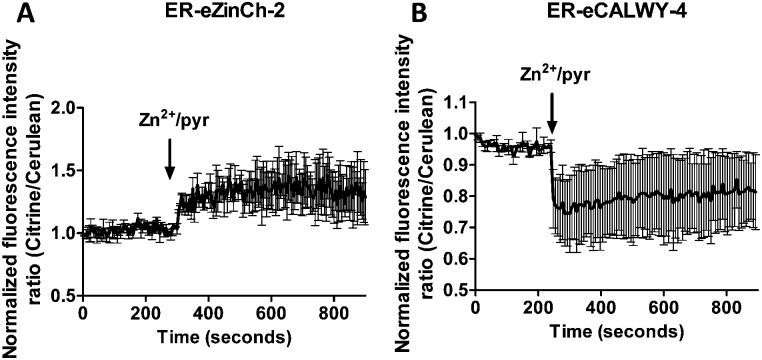
(A) Responses of MCF-7 cells expressing ER-eZinCh-2 to the addition of 20 μM Zn^2+^/10 μM pyrithione. (B) Responses of TamR cells expressing ER-eCALWY-4 to the addition of 20 μM Zn^2+^/10 μM pyrithione. Traces in A and B represent the average of three cells after normalization at *t* = 0 s. Error bars represent SEM.

This fast increase in fluorescence is consistent with direct pyrithione-mediated transfer of Zn^2+^ from the cell exterior into the cytoplasm, without the need to invoke intracellular release of Zn^2+^ from the ER. To directly assess the effect of stimulation with Zn^2+^/pyrithione on ER Zn^2+^ levels, we repeated these experiments in MCF-7 and TamR cells expressing either ER-ZinCh-2 or ER-eCALWY-4. Addition of 20 μM Zn^2+^/10 μM pyrithione to MCF-7 cells expressing ER-eZinCh-2 resulted in an immediate increase in emission ratio (Fig. 5A), consistent with an increase in the free ER Zn^2+^ concentration. Comparable responses were observed for TamR cells expressing ER-eZinCh-2 (Fig. S1, ESI†). The same experiments were performed on both MCF-7 (Fig. S2, ESI†) and TamR (Fig. 5B) cells expressing ER-eCALWY-4, resulting in a decrease in emission ratio upon Zn^2+^ addition, which again corresponds to an increase in free ER Zn^2+^ levels. The increase in ER Zn^2+^ is inconsistent with a specific release of Zn^2+^ from the ER to the cytoplasm, but is not unexpected given the fact that pyrithione can also mediate transfer of Zn^2+^ across the intracellular ER membrane.

### Monitoring changes in intracellular Zn^2+^ upon stimulation with EGF and ionomycin

**Fig. 6:**
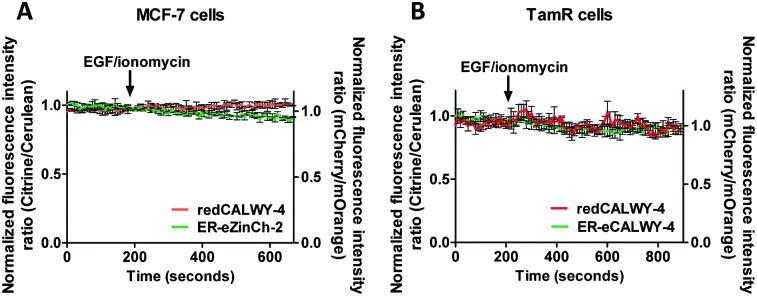
(A) Responses of MCF-7 cells co-expressing redCALWY-4 and ER-eZinCh-2 to the addition of 10 ng mL^–1^ EGF together with 500 nM ionomycin. (B) Responses of TamR cells co-expressing redCALWY-4 and ER-eCALWY-4 to the addition of 10 ng mL^–1^ EGF together with 500 nM ionomycin. Traces in A and B represent the average of three cells after normalization at *t* = 0 s. Error bars represent SEM.

A combination of epidermal growth factor (EGF) and the calcium ionophore ionomycin has been reported previously to trigger release of Zn^2+^ from the ER resulting in so-called Zn^2+^ waves in the cytosol of mast cells.^3^ Using the synthetic Zn^2+^ probe Zinquin (*K*_d_ = 7 μM), a comparable increase in cytosolic Zn^2+^ concentration has been reported in TamR cells upon addition of EGF and ionomycin.^21^ To obtain independent evidence for the putative release of Zn^2+^ from the ER into the cytosol upon EGF/ionomycin stimulation, we simultaneously used redCALWY-4 to monitor cytosolic Zn^2+^ levels and either ER-eZinCh-2 or ER-eCALWY-4 to monitor changes in ER Zn^2+^ concentrations.^10^ Both cell lines showed good co-expression levels of these proteins. Imaging experiments were again performed using a perfusion setup, starting with perfusion of imaging buffer followed by addition of buffer supplemented with 10 ng mL^–1^ EGF and 500 nM ionomycin (Fig. 6). When Zn^2+^ would be released from the ER to the cytosol upon external stimulation, a decrease in the emission ratio of redCALWY-4 and ER-eZinCh-2 should be observed, while an increase in emission ratio would be expected for ER-eCALWY-4. However, no changes in emission ratio were observed upon perfusion with EGF and ionomycin in either the cytosol or the ER (Fig. 6). Different combinations of sensors were tested for both wild-type MCF-7 and TamR cells, but none of these experiments revealed a detectable change in either cytosolic or ER Zn^2+^ levels.

**Fig. 7:**
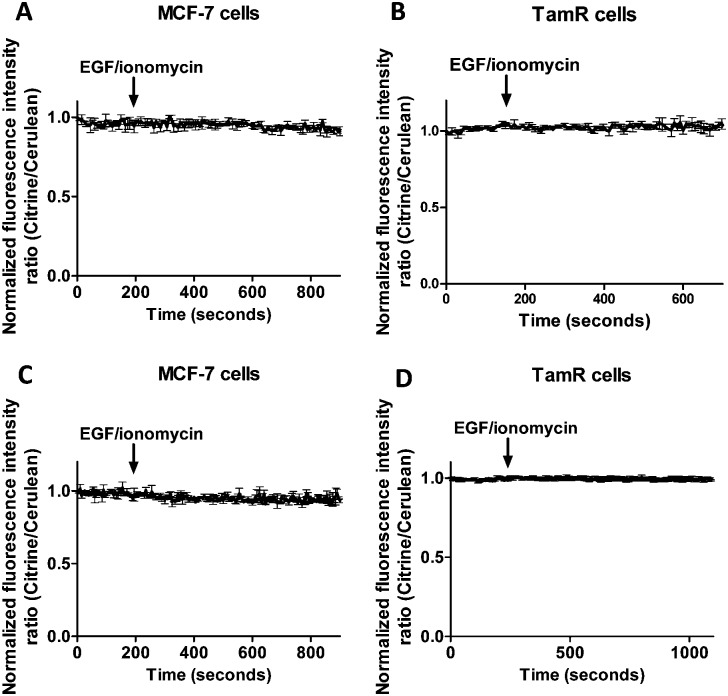
Responses of MCF-7 cells expressing cytosolic eCALWY-4 (A) and TamR cells expressing cytosolic eZinCh-2 (B) to the addition of 10 ng mL^–1^ EGF together with 500 nM of the calcium ionophore ionomycin. Responses of MCF-7 cells expressing ER-eZinCh-2 (C) and TamR cells expressing ER-eCALWY-4 (D) to the addition of 10 ng mL^–1^ EGF together with 500 nM of the calcium ionophore ionomycin. All traces represent the average of three cells after normalization at *t* = 0 s. Error bars represent SEM.

To exclude the possibility that the lack of response to EGF/ionomycin was somehow a result of the multicolor imaging experiments, we repeated these experiments in single sensor experiments. In these experiments the classical cerulean/citrine-based probes can be used for cytosolic imaging. The higher dynamic range of these sensors compared to the redCALWY probes should make it easier to detect small changes in cytosolic Zn^2+^ levels. However, no changes in emission ratio were observed following addition of EGF and ionomycin in MCF-7 cells (Fig. 7A) or TamR cells (Fig. 7B) transfected with either eZinCh-2 or eCALWY-4. Similarly, EGF/ionomycin addition did not provoke any sensor response in either MCF7 or TamR cells expressing ER-eZinCh-2 or ER-eCALWY-4 (Fig. 7C and D).

## Discussion and conclusion

The previously developed FRET-based Zn^2+^ sensors eZinCh-2 and eCALWY-4 were successfully used to test the hypothesis that exposure of wild-type MCF-7 and TamR cells to external stimuli results in ZIP7-dependent Zn^2+^ release from the ER stores into the cytosol.^21^ Our work provided the first accurate determination of the free Zn^2+^ concentrations in these breast cancer cell lines and revealed that free Zn^2+^ concentrations are similar to those reported previously in other cell lines, both in the cytosol and the ER.^6^ In contrast to previous reports using a synthetic fluorescent sensor and fixed cells, the genetically encoded sensors showed that addition of external Zn^2+^ and the Zn^2+^-specific ionophore pyrithione results in an immediate increase in both cytosolic and ER Zn^2+^. This result is important, because external Zn^2+^/pyrithione was previously hypothesized to induce an intracellular signaling cascade resulting in ZIP7-mediated release of Zn^2+^ from the ER to the cytosol. Another treatment that had been implicated to trigger specific release of Zn^2+^ from the ER to the cytosol, EGF and ionomycin, was extensively tested in live cell imaging experiments in cells expressing a cytosolic sensor, an ER-targeted sensor or a combination of both. No changes in emission ratio were observed in either the cytosol or the ER in either MCF7 cells or TamR cells. These results suggest that EGF/ionomycin treatment does not trigger Zn^2+^ release from the ER into the cytosol.

An important difference between this work and previous reports is the use of genetically encoded sensors instead of small-molecule dyes. Using the Zn^2+^-specific fluorescent dye FluoZin-3, Taylor and coworkers did not observe an immediate increase in fluorescence after the addition of Zn^2+^/pyrithione, which is surprising because pyrithione is a Zn^2+^-specific ionophore that transfers Zn^2+^ across the plasma membrane. One possible explanation is that the fixation protocol that was used previously interfered with accurate and early detection of increases in Zn^2+^ concentration. *E.g.* fixation does not allow continuous monitoring of the free Zn^2+^ concentration in the same cell and cells on different coverslips were required for each time point after Zn^2+^ treatment. Finally, unlike genetically encoded FRET sensors that can easily be targeted to a specific place in the cell, FluoZin-3 has been shown to not be exclusively localized in the cytosol, but also be present in other subcellular compartments.^23^

In contrast to previous studies using Zinquin, no detectable changes in emission ratio were observed using our genetically encoded sensors in either MCF-7 or TamR cells following addition of EGF/ionomycin, neither in the cytosol nor when the sensors were targeted to the ER. Because the affinity of Zinquin that was used in previous studies is relatively low (*K*_d_ = 7 μM), increasing levels of cytosolic Zn^2+^ upon EGF/ionomycin treatment should have been easily detectable by the sensors used in this study, eZinCh-2 (*K*_d_ = 1 nM at pH 7.1) and eCALWY-4 (*K*_d_ = 0.63 pM at pH 7.1) (Table 1). We therefore conclude that addition of external EGF together with ionomycin does not trigger release of Zn^2+^ from the ER stores into the cytosol. Previous experiments with EGF/ionomycin were also performed on fixed cells, which holds the same limitations as discussed above. It may therefore be worthwhile to apply genetically encoded sensors also in other cell systems in which treatment with EGF/ionomycin has been reported to result in a transient increase in cytosolic Zn^2+^ levels such as the Zn^2+^ waves reported by Yamasaki and coworkers in mast cells.^3^

Having shown that addition of Zn^2+^ together with pyrithione results in an immediate increase in cytosolic and ER free Zn^2+^ concentrations, our results do not support the suggested Zn^2+^ release from the ER several minutes after this treatment. In addition, no changes in overall cytosolic or ER free Zn^2+^ levels were observed upon addition of EGF/ionomycin using our genetically encoded sensors. Other observations that were originally linked to the release of Zn^2+^ from the ER may therefore also need to be reconsidered. For example, the association of protein kinase CK2α with ZIP7, and the subsequent phosphorylation of ZIP7, could be a downstream consequence of the pyrithione-mediated increase in cytosolic Zn^2+^. Similarly, the reported activation of downstream signaling pathways may occur through phosphatase inhibition.^20^ Addition of 20 μM Zn^2+^ together with 10 μM pyrithione will increase the level of cytosolic free Zn^2+^ level to at least several nanomolar, which is sufficient to directly inhibit phosphatases.^25^ Since no changes in overall intracellular Zn^2+^ levels were observed upon treatment with EGF/ionomycin, another mechanism may need to be invoked to explain the increased levels of tyrosine phosphorylation observed after treatment with this trigger. Alternatively, phosphatase inhibition may result from transient increases in local Zn^2+^ concentrations that cannot be detected using the current sensor technology.

In conclusion, the present study using genetically encoded FRET sensors does not provide supportive evidence for ZIP7-mediated release of Zn^2+^ from ER stores into the cytosol in TamR cells. These unexpected results suggest that it may be worthwhile to apply the specific properties of genetically encoded FRET sensors to carefully reassess other examples of triggered Zn^2+^ release from intracellular stores.

## Experimental section

### Mammalian cell culture and transfection

MCF-7 and TamR cells were cultured in Roswell Park Memorial Institute media (RPMI), supplemented with 5% (v/v) fetal calf serum (FCS), 2 mM glutamine, 1 mM fungizone, 100 U mL^–1^ penicillin, and 100 μg mL^–1^ streptomycin (all from Life Technologies) at 37 °C in a humidified atmosphere containing 5% CO_2_. For TamR cells stripped fetal calf serum (SFCS) was used instead of FBS and 0.1 μM 4-hydroxytamoxifen (4-OH tamoxifen) was added to the media. Cells were seeded on glass coverslips (*φ* 30 mm, VWR) one day before transfection. About 200 000 cells were seeded to reach a confluency of ∼80% on the day of transfection. Lipofectamine 2000 (Life Technologies) was used to carry out transfections, following the manufacturer's instructions. Cells were imaged either one day (single sensor experiments) or two days (two sensor experiments) after transfection in a HEPES buffer (Live Cell Imaging Buffer, Life Technologies) at 37 °C.

### Intracellular FRET imaging

Imaging on MCF-7 and TamR cells was performed with a confocal microscope (Leica, TCS SP5X) equipped with a 63× water immersion objective, acousto-optical beamsplitters (AOBS), a white light laser, and a 405 nM laser. Fluozin-3 AM was excited at 494 nm, followed by monitoring its emission between 505–530 nm. For all eCALWY-4 and eZinCh-2 constructs, cerulean was excited using the 405 nm laser. For the redCALWY-4 the emission of a white light laser was set to 550 nm (5% of full power) to excite mOrange2. Emission was monitored using the AOBS and avalanche photo diode/photomultiplier tubes hybrid detectors (HyD, Leica): cerulean (450–500), citrine (515–595 nm), mOrange2 (565–600), and mCherry (600–630). Images were recorded at either 7.5 s intervals (two sensor experiments) or at 5 s intervals (single sensor experiments). For the *in situ* calibration experiments, cells were imaged for a few minutes with HEPES buffer without additives. Next HEPES buffer containing 50 μM *N*,*N*,*N*′,*N*′-tetrakis(2-pyridylmethyl)ethylenediamine (TPEN, Sigma) was added, followed by addition of HEPES buffer containing 100 μM ZnCl_2_ and 5 μM of the Zn^2+^-specific ionophore 2-mercaptopyridine *N*-oxide (pyrithione, Sigma), with a final concentration of 36 μM TPEN, 100 μM ZnCl_2_ and 5 μM pyritione. Zn^2+^ stimulation was performed by perfusion with plain HEPES buffer for a few minutes, followed by perfusion with HEPES buffer supplemented with 20 μM ZnCl_2_ and 10 μM pyrithione (Sigma). For EGF/ionomycin stimulation, cells were perfused with HEPES buffer without additives for a few minutes, followed by perfusion with HEPES buffer containing 10 ng mL^–1^ EGF (Sigma, E9644) and 500 nM ionomycin (Sigma, 56092-82-1). Image analysis was performed using ImageJ software as described before.^24,26^ Steady-state fluorescence intensity ratio of acceptor over donor was measured, followed by the determination of the minimum and maximum ratios to calculate the free Zn^2+^ concentration using eqn (1):1
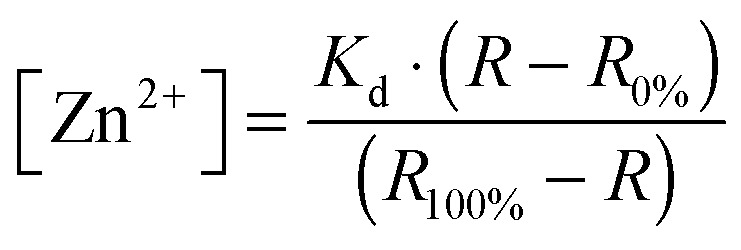
 In which *R*_0%_ is the ratio in the Zn^2+^ depleted state, after addition of 50 μM TPEN, and *R*_100%_ was obtained upon Zn^2+^ saturation with 100 μM ZnCl_2_ in the presence of 5 μM pyrithione.^6,24^

## References

[cit1] Vallee B. L., Auld D. S. (1990). Biochemistry.

[cit2] Truong-Tran A. Q., Carter J., Ruffin R. E., Zalewski P. D. (2001). BioMetals.

[cit3] Yamasaki S., Sakata-Sogawa K., Hasegawa A., Suzuki T., Kabu K., Sato E., Kurosaki T., Yamashita S., Tokunaga M., Nishida K., Hirano T. (2007). J. Cell Biol..

[cit4] Yamashita S., Miyagi C., Fukada T., Kagara N., Che Y. S., Hirano T. (2004). Nature.

[cit5] Hogstrand C., Kille P., Nicholson R. I., Taylor K. M. (2009). Trends Mol. Med..

[cit6] Vinkenborg J. L., Nicolson T. J., Bellomo E. A., Koay M. S., Rutter G. A., Merkx M. (2009). Nat. Methods.

[cit7] Qin Y., Dittmer P. J., Park J. G., Jansen K. B., Palmer A. E. (2011). Proc. Natl. Acad. Sci. U. S. A..

[cit8] Hessels A. M., Merkx M. (2015). Metallomics.

[cit9] Zhao L., Oliver E., Maratou K., Atanur S. S., Dubois O. D., Cotroneo E., Chen C. N., Wang L., Arce C., Chabosseau P. L., Ponsa-Cobas J., Frid M. G., Moyon B., Webster Z., Aldashev A., Ferrer J., Rutter G. A., Stenmark K. R., Aitman T. J., Wilkins M. R. (2015). Nature.

[cit10] Lindenburg L. H., Hessels A. M., Ebberink E. H., Arts R., Merkx M. (2013). ACS Chem. Biol..

[cit11] Hessels A. M., Chabosseau P., Bakker M. H., Engelen W., Rutter G. A., Taylor K. M., Merkx M. (2015). ACS Chem. Biol..

[cit12] Palmiter R. D., Huang L. (2004). Pflugers Arch.

[cit13] Taylor K. M., Nicholson R. I. (2003). Biochim. Biophys. Acta.

[cit14] Taylor K. M., Morgan H. E., Johnson A., Hadley L. J., Nicholson R. I. (2003). Biochem. J..

[cit15] Suzuki A., Endo T. (2002). Gene.

[cit16] Santoliquido P. M., Southwick H. W., Olwin J. H. (1976). Surg., Gynecol. Obstet..

[cit17] Margalioth E. J., Schenker J. G., Chevion M. (1983). Cancer.

[cit18] Nandi S., Guzman R. C., Yang J. (1995). Proc. Natl. Acad. Sci. U. S. A..

[cit19] Woo W., Xu Z. (2002). Biol. Trace Elem. Res..

[cit20] Haase H., Maret W. (2005). J. Trace Elem. Med. Biol..

[cit21] Taylor K. M., Hiscox S., Nicholson R. I., Hogstrand C., Kille P. (2012). Sci. Signaling.

[cit22] Taylor K. M., Vichova P., Jordan N., Hiscox S., Hendley R., Nicholson R. I. (2008). Endocrinology.

[cit23] Qin Y., Miranda J. G., Stoddard C. I., Dean K. M., Galati D. F., Palmer A. E. (2013). ACS Chem. Biol..

[cit24] Chabosseau P., Tuncay E., Meur G., Bellomo E. A., Hessels A., Hughes S., Johnson P. R., Bugliani M., Marchetti P., Turan B., Lyon A. R., Merkx M., Rutter G. A. (2014). ACS Chem. Biol..

[cit25] Maret W., Jacob C., Vallee B. L., Fischer E. H. (1999). Proc. Natl. Acad. Sci. U. S. A..

[cit26] Schneider C. A., Rasband W. S., Eliceiri K. W. (2012). Nat. Methods.

