# Supplementary material for: Monitoring cytosolic and ER Zn2+ in stimulated breast cancer cells using genetically encoded FRET sensors
Source: Metallomics. 2016 Jan 7;8(2):211–7. doi: 10.1039/c5mt00257e (PMC4756312; doi:10.1039/c5mt00257e)
Supplement: Supplementary file 1 [file MT-008-C5MT00257E-s001.pdf]

Electronic Supplementary Material (ESI) for Metallomics.
This journal is © The Royal Society of Chemistry 2015

Supplementary Information

Monitoring Cytosolic and ER Zn^{2+} in Stimulated Breast Cancer Cells using Genetically Encoded FRET Sensors

Anne M. Hessels[a], Kathryn M. Taylor[b], and Maarten Merkx[a]*

[a]Laboratory of Chemical Biology and Institute of Complex Molecular Systems (ICMS), Department of Biomedical Engineering, Eindhoven University of Technology, Eindhoven, The Netherlands. [b] Breast Cancer Molecular Pharmacology Group, School of Pharmacy and Pharmaceutical Sciences, Cardiff University, Cardiff, UK.

* corresponding author: m.merkx@tue.nl

Supplementary figures

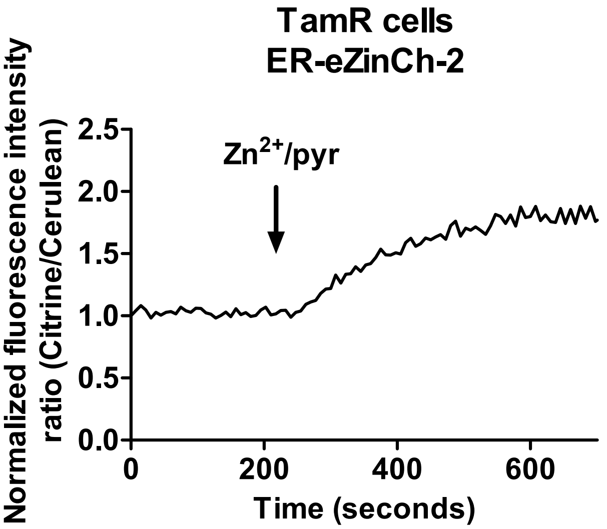

Figure S1: Response of TamR cell expressing ER-eZinCh-2 to the addition of 20 µM Zn^{2+}/10 µM pyrithione.

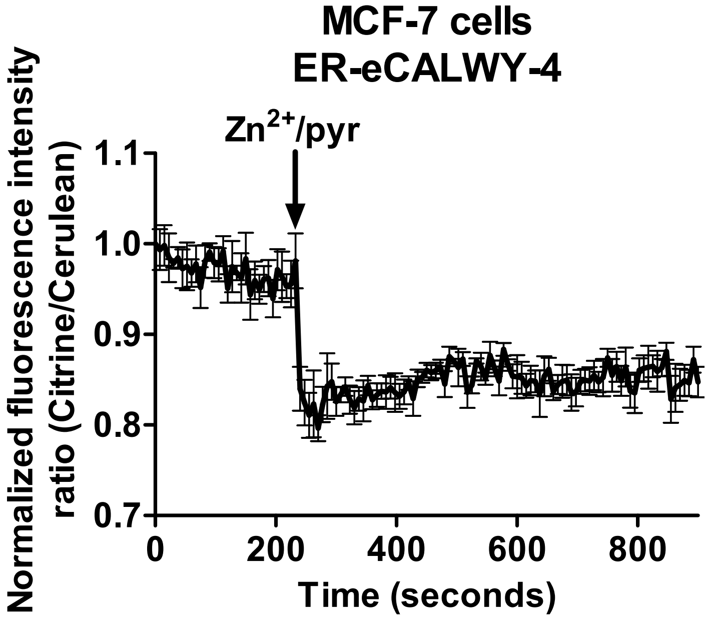

Figure S2: Responses of MCF-7 cells expressing ER-eCALWY-4 to the addition of 20 µM Zn^{2+}/10 µM pyrithione. Traces represent the average of three cells after normalization at t=0 s. Error bars represent SEM.
